# 

**Published:** 1868-12

**Authors:** 


### CONTENTS.

ORIGINAL COMMUNICATIONS.

The Life of the Trichina ........ 487

Report of Discussions at the Ohio State Dental Association ........ 501

Microscopy of the Teeth ........ 521

EDITORIAL.

Ohio State Dental Association ........ 526
Examinations ........ 526
A Practical Treatise on Mechanical Dentistry ........ 528
American Dental Association—Notice ........ 528
Close of the volume ........ 529

